# Does open reduction internal fixation using a volar locking plate and closed reduction percutaneous pinning using K wires provide similar functional and radiological outcomes for unstable distal radius fractures?

**DOI:** 10.1051/sicotj/2023015

**Published:** 2023-06-23

**Authors:** Ahmad Radaideh, Jehad Abualadas, Yazan Anaqreh, Adham Alnemer, Ahmad Abdalmajeed Alghzawi, Rawan Abualadas, Mohammad Alawneh, Suhaib Bani Essa

**Affiliations:** 1 Department of Special Surgery, Orthopaedic division, Faculty of Medicine, Jordan University of Science and Technology Irbid 22110 Jordan; 2 Department of Public Health, Faculty of Medicine, Jordan University of Science and Technology Irbid 22110 Jordan; 3 Faculty of Medicine, Jordan University of Science and Technology Irbid 22110 Jordan

**Keywords:** Distal radius fracture (DRF), Open reduction internal fixation (ORIF), Closed reduction percutaneous pinning (CRPP)

## Abstract

*Background*: Distal radius fractures (DRFs) are a common orthopedic injury, with open reduction internal fixation (ORIF) and closed reduction percutaneous pinning (CRPP) being the two most frequently used methods for treating unstable DRFs. The optimal treatment approach for DRFs is still a matter of debate. Therefore, this retrospective analysis aimed to compare the functional and radiological outcomes of ORIF and CRPP to determine the most effective approach for treating unstable DRFs. *Material and Methods*: A total of 89 patients were included in this retrospective study; 34 underwent CRPP and 55 underwent ORIF (61 males and 28 females, mean age: 35.5). Radiographic measurements of radial inclination, radial height, and volar tilt, as well as patient-rated wrist evaluation (PRWE) scores for pain and function, were used to evaluate the functional and radiological outcomes during the final follow-up period, ranging from 1 to 4 years. *Results*: There were significant improvements in the radiographic measurements for both groups, indicating a good reduction. However, the two fixation methods had no significant difference in radiographic measurements during the entire follow-up period. Regarding PRWE scores, there was a significant difference between the two groups, with patients in the CRPP group reporting better wrist function and less pain. *Conclusions*: Both CRPP and ORIF are effective methods for treating unstable DRFs. Achieving an acceptable reduction did not correlate with better pain management, function, or the ability to carry out day-to-day activities. However, patients treated with CRPP had better wrist function and less pain during follow-up. Radiographic measurements did not differ significantly between the two groups. Clinicians should consider closed-reduction percutaneous pinning as a viable and effective treatment option for distal radius fractures, particularly when optimal wrist function and pain management are important considerations.

## Introduction

Distal radius fractures (DRFs) are a common type of injury, accounting for 16% of all fractures that are brought to emergency services The incidence of DRFs increases with aging and increasing numbers of high-energy injuries such as falls and car accidents, and the intra-articular type accounts for approximately a quarter of all distal radius fractures [1, 2].

Treatment options for DRFs are numerous and depend on various factors, such as the type of fracture and patient characteristics. These options can include conservative and surgical approaches with closed or open reduction and internal or external fixation methods. The decision-making process for selecting the appropriate treatment method involves taking a thorough history from the patient, performing a comprehensive physical examination, and critically evaluating appropriate radiographs, as well as the surgeon’s preference [3, 4].

Both closed reduction with percutaneous pinning (CRPP) and open reduction with internal fixation (ORIF) are considered viable treatment options [3]. However, it is difficult to determine which of the two fixation methods is superior [4]. While Rozental et al. [5] reported that both groups of patients who underwent ORIF and CRPP achieved similar results at the end of the first year, the positive and negative aspects of each method have not yet been conclusively established [6–9].

Although there is no standard protocol for dealing with DRFs, restoring normal anatomy is always the primary focus of treatment. Closed techniques can be used to treat stable, reducible DRFs [10], but in most cases of unstable distal radius fractures, it is not possible to restore distal radius integrity using closed techniques. In these situations, open or percutaneous surgical techniques are required [11, 12]. Despite some studies comparing the outcomes of ORIF and CRPP for DRFs, there is still no consensus on which approach is superior. Additionally, few studies have explored the relationship between radiological parameters and clinical outcomes in this context. Therefore, the relationship between radiological parameters and clinical outcomes in this context. Therefore, this study aims to compare the radiological and clinical outcomes of ORIF with volar plating and CRPP with k-wires for DRFs, with a particular focus on examining the connections between radiological parameters and clinical outcomes.

## Material and methods

Our retrospective cohort study was conducted at the Orthopedic Department of King Abdullah University Hospital (KAUH), Jordan. Prior to beginning this investigation, approval was granted by an Institutional Review Board (IRB) committee (IRB approval number 26/148/2022, dated April 13, 2022).

In this study, the inclusion criteria were patients who had a distal radius fracture with the instability that was treated by either ORIF or CRPP at the Orthopedic Department of King Abdullah University Hospital (KAUH), Jordan, between January 2015 and December 2021. On the other hand, the exclusion criteria were patients with distal radius fractures with accompanying injuries, systemic inflammatory arthritis, neuromuscular disorders, open fractures, or previous DRF. Patients with incomplete medical records or those who were lost to follow-up were also excluded from the study.

**Figure 1:**
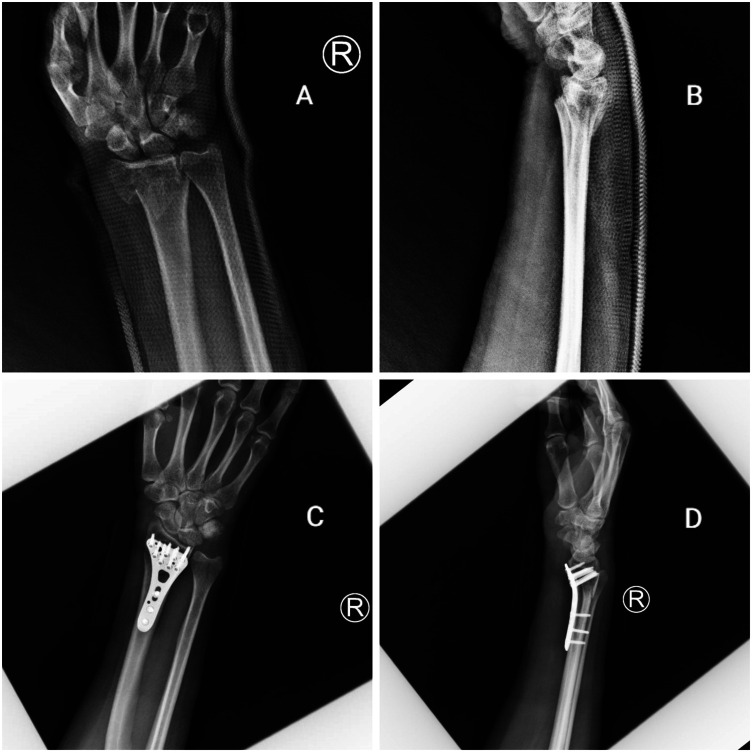
(A) Pre-operative distal radius fracture AP view; (B) pre-operative distal radius fracture lateral view; (C) post-operative ORIF distal radius fracture AP view (D) post-operative ORIF distal radius fracture lateral view.

**Figure 2:**
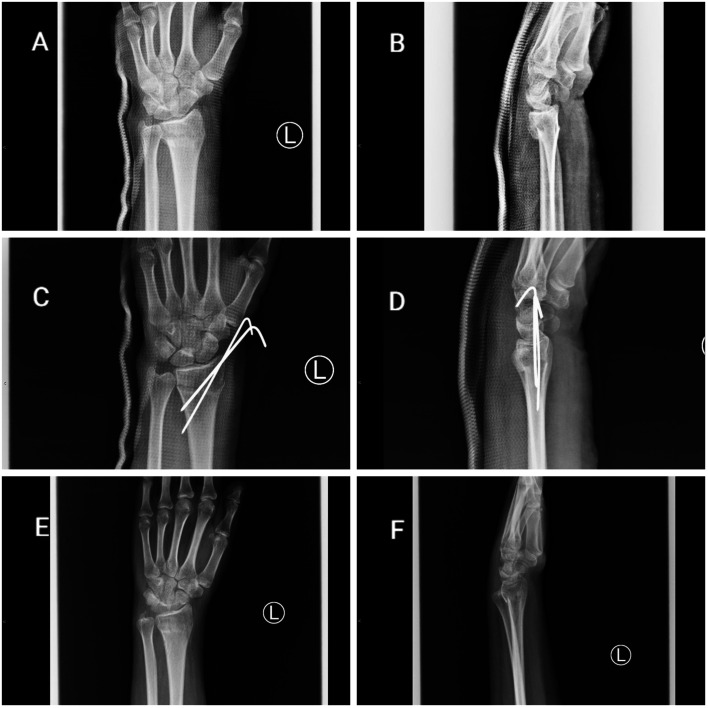
(A) Pre-operative distal radius fracture AP view; (B) pre-operative distal radius fracture lateral view; (C) post-operative percutaneous pinning of distal radius fracture AP view; (D) post-operative percutaneous pinning of distal radius fracture lateral view; (E) last follow-up distal radius fracture AP view; (F) last follow-up distal radius fracture lateral view.

This study reviewed a total of 89 patients with unstable DRFs who were presented to our institution (KAUH) between January 2015 and December 2021 and treated by reduction and fixation using ORIF and CRPP (61 males and 28 females, mean age: 35.5 years old). The patients were divided into two groups: 55 patients in the ORIF group (34 males and 21 females, mean age: 39.51 years old) and 34 patients in the CRPP group (27 males and 7 females, mean age: 29.1 years old), and the clinical outcomes were assessed 1–4 years after surgery as the primary endpoint; the average follow-up period was nearly three years. Distal radius fractures can be classified in a number of ways. They have been developed to guide the treatment plan and predict the prognosis of patients. In the fields of research and science, the AO classification has been adopted and used [13]. The fracture type according to the AO classification on preoperative plain X-ray images was A in 8 and B and C in 47 patients in the ORIF group, and A in 21 and B and C in 13 patients in the CRPP group (Figures 1 and 2).

Patients were assigned to the ORIF or CRPP group based on the surgeon’s preference and fracture characteristics. ORIF procedures were performed using the modified Henry approach with locking plates, while CRPP procedures involved closed reduction and pinning with K-wires. The surgical techniques used were consistent across all patients in each group. The average duration between the fracture and the time of surgery was 48 h.

Two trained research assistants independently reviewed the medical records of eligible patients to extract radiographic data. Discrepancies were resolved by consensus between the two reviewers. Relevant data was then entered into a password-protected database for further analysis.

Clinical hand function was evaluated using the patient-rated wrist evaluation score. The PRWE is a questionnaire designed to measure wrist pain and disability [14]. The PRWE consists of two subscales: the pain subscale, which contains five items, and the function subscale, which contains a total of 10 items. The maximum score in each section is 50, and the minimum is 0. The overall score is determined by adding up the values for both pain and function, which range from 0 to 100, with 0 signifying the best possible outcome. A better outcome corresponds to lower score. During the most recent follow-up check, a comparison was made between the two groups regarding pain and function throughout both specialized and typical activities.

Imaging was performed using standard radiographic techniques, including anteroposterior and lateral views of the affected wrist. All patients had their X-rays taken with the wrist in a neutral position, the elbow flexed at 90 degrees, and the shoulder in a neutral position. The X-rays were evaluated by two independent orthopedic surgeons who were blinded to the treatment group. To ensure accuracy and consistency in the radiographic data measurements, two orthopedic surgeons independently reviewed the radiographs. Randomly selected cases were remeasured by a third orthopedic surgeon who was not involved in the initial measurements. The radiographic measurements were performed on digital radiographs using standard techniques, including measurements of radial length, inclination, and tilt, according to the AO classification system [13]. Statistical analysis was performed using IBM SPSS Statistical Software (version 28). According to the data type, we present the data as the mean (SD), numbers, or percentages, as appropriate. The mean values (with standard deviations) were used to present the radiological measurements and the patient-rated wrist evaluation (PRWE) score. Independent t-tests and chi-square tests were used to compare the two groups as appropriate. The significance level was set at 0.05.

## Results

The study sample included 89 patients, divided into two groups based on the method of fixation; Table 1 illustrates the basal demographic characteristics of both groups. The ORIF group was significantly older and had a higher percentage of B–C fractures compared to the percutaneous pinning group. There were no significant differences between the two groups regarding gender or smoking status.

**Table 1:** Demographic and preoperative characteristics.

	ORIF	Pinning	*P*-value	Total
No. of patients	55 (61.8%)	34 (38.2%)		89
Mean age at time of fracture*	39.51 (11.74)	29.1 (15.93)	0.002	35.52 (14.35)
Mean age at follow-up	42.67 (11.94)	31.74 (16.6)	0.001	38.5 (14.81)
Follow-up period	3.16 (1.71)	2.68 (1.77)	0.201	2.98 (1.74)
Smoker**	16 (18%)	4 (4.5%)	0.057	20 (22.5%)
Gender**				
Male	34 (38.2%)	27 (30.3%)	0.082	61 (68.5%)
Female	21 (23.6%)	7 (7.9%)		28 (31.5%)
Fracture type**				
A	8 (9%)	21 (23.6%)	0.0001	29 (32.6%)
B + C	47 (52.8%)	13 (14.6%)		60 (67.4%)

* Standard deviations and mean values are provided.

** The values are given as the number of patients, with the percentage of the total number of patients in the study group (*N* = 89).

The pre-operative to post-operative radiologic measurements were observed in both groups separately (Table 2) and the post-operative radiological measurements of volar tilt, radial height, and radial inclination showed significant improvement from the preoperative measurement, indicating proper reduction.

**Table 2:** Radiographic measurements and PRWE score.

	ORIF	Pinning	*p*-Value
Volar tilt	9.82 (6.96)	9.15 (5.52)	0.635
Radial height	11.65 (2.58)	11.7 (3.31)	0.935
Radial inclination	20.35 (4.23)	20.56 (4.2)	0.817
PRWE score pain	24.5 (11.39)	14.94 (13.59)	0.001
PRWE Score function	23.41 (13.39)	14.9 (16.24)	0.009
PRWE score total	48.15 (22.3)	29.85 (28.98)	0.003

The values are given as the average, with the standard deviation.

A comparison was made between the two groups based on postoperative radiological measurements and the PRWE score (total score, pain score, and function score) (Table 3). Both methods of fixation did not show any significant difference regarding the postoperative radiological measurements (volar tilt, radial height, and radial inclination). However, patients who underwent the ORIF type of fixation had a significantly higher PRWE score (pain and function) compared to patients who underwent the pinning type of fixation.

**Table 3:** Radiographic measurements preoperative and postoperative.

	ORIF	Pinning	Total						
	PRE-Operative	POST-Operative	*P*-value	Pre-Operative	Post-Operative	P-value	Pre-Operative	Post-Operative	*P*-value
Volar tilit	−2.16 (11.63)	9.82 (6.96)	< 0.0001	0.97 (8.85)	9.82 (6.96)	< 0.0001	−0.97 (10.71)	9.56 (6.42)	< 0.0001
Radial height	7.35 (4.29)	11.65 (2.58)	< 0.0001	8.09 (4.57)	11.7 (3.31)	< 0.0001	7.63 (4.39)	11.67 (2.86)	< 0.0001
Radial inclination	13.58 (5.26)	20.35 (4.23)	< 0.0001	13.29 (6.41)	20.56 (4.2)	< 0.0001	13.47 (5.69)	20.43 (4.2)	< 0.0001

The values are given as the average, with the standard deviation.

## Discussion

Distal radius fracture (DRF) are among the most common injuries seen in orthopedic practices [15], and the optimal surgical treatment for unstable displaced fractures remains a topic of debate. In this study, we compared two surgical procedures, ORIF and CRPP, to investigate their clinical and radiological outcomes for treating unstable distal radius fractures. Our findings showed that the clinical outcome was better for the CRPP group, while the radiological outcome was similar for both methods. Despite the lack of evidence to guide orthopedic surgeons in this area, there has been a significant shift in the treatment of unstable distal radius fractures toward the use of locking plates. This shift has occurred despite the fact that there is no evidence to inform orthopedic surgeons in this area [4].

This study has several limitations that could affect clinical practice. The study only included a limited number of patients as participants, and we had a follow-up average of between 12 months and 4 years after surgery. Longer-term outcomes, such as the development of wrist arthritis, require more extensive follow-up. However, there are only a limited number of reported studies comparing clinical and radiological outcomes between ORIF and CRPP.

Our study suggests that CRPP is a safe, easy, and successful method for treating unstable distal radius fractures, both extraarticular and intraarticular, simple and complex. Percutaneous pinning is biomechanically stiff, although there is not enough evidence to justify it. Open reduction and internal fixation are advised for two types of fractures: Barton fractures and complicated intra-articular fractures [16]. Previous studies have also investigated the use of ORIF for treating distal radius fractures. For instance, Jupiter et al. [17] found that ORIF resulted in good results in the majority of the patients who had displaced colles type DRF and recommended ORIF as a treatment for older patients who had redisplaced distal radial fractures. Beharrie et al. [18] suggested that using ORIF for elderly patients with unstable DRF is a safe and effective modality of treatment.

Another study was conducted by Rozental et al. [5]. They assessed patients who underwent both modalities of fixation (ORIF and CRPP) and stated that the ORIF group achieved better functional results in the first 12 weeks of follow-up, but both groups (ORIF and CRPP) achieved similar results at the end of the first year.

In our study, the PRWE score was statistically better in the pinning group at the end of an average follow-up of 3 years, which is similar to the results reported by Kreder et al. [19]. They found no significant difference in the radiological restoration of anatomical features between CRPP and ORIF in patients with intraarticular distal radius fractures. However, there was a better functional outcome in the CRPP group when the intra-articular step and gap were minimized. In many studies, ORIF was better at radiological alignment restoration; however, there was no significant difference in Kreder et al.’s study.

According to the results of our study, radiographic alignment (volar tilt, radial inclination, and radial height) was statistically not significant between the two treatment groups. The distal radius appears to be relatively tolerant to changes in volar tilt, radial inclination, and radial height with no apparent functional deficits, and radiographic measurement changes will not consistently translate into poorer clinical outcomes [20]. Our research suggests that a reasonable reduction was achieved by the two methods of fixation, and we found that radiographic measurements were not related to improved function.

## Conclusion

In conclusion, our study provides further evidence that both ORIF and CRPP are viable options for treating unstable distal radius fractures. At the final follow-up, the level of pain and function indicated by the PRWE scores was better in the pinning group, and radiographic measurements were similar between the two treatment groups for unstable distal radius fractures.
